# Advances in the Application of Natural Products and the Novel Drug Delivery Systems for Psoriasis

**DOI:** 10.3389/fphar.2021.644952

**Published:** 2021-04-21

**Authors:** Jin Xie, Shengjie Huang, Haozhou Huang, Xuan Deng, Pengfei Yue, Junzhi Lin, Ming Yang, Li Han, Ding-kun Zhang

**Affiliations:** ^1^ State Key Laboratory of Southwestern Chinese Medicine Resources, Pharmacy School, Chengdu University of Traditional Chinese Medicine, Chengdu, China; ^2^ State Key Laboratory of Innovation Medicine and High Efficiency and Energy Saving Pharmaceutical Equipment, Jiangxi University of Traditional Chinese Medicine, Nanchang, China; ^3^ TCM Regulating Metabolic Diseases Key Laboratory of Sichuan Province, Hospital of Chengdu University of Traditional Chinese Medicine, Chengdu, China

**Keywords:** psoriasis, pathogenesis, natural products, novel drug delivery systems, target

## Abstract

Psoriasis, an incurable autoimmune skin disease, is one of the most common immune-mediated disorders. Presently, numerous clinical research studies are underway, and treatment options are available. However, these treatments focus on improving symptoms of the disease and fail to achieve a radical cure; they also have certain toxic side effects. In recent years, natural products have increasingly gained attention because of their high efficiency and low toxicity. Despite their obvious therapeutic effects, natural products’ biological activity was limited by their instability, poor solubility, and low bioavailability. Novel drug delivery systems, including liposomes, lipospheres, nanostructured lipid carriers, niosomes, nanoemulsions, nanospheres, microneedles, ethosomes, nanocrystals, and foams could potentially overcome the limitations of poor water solubility and permeability in traditional drug delivery systems. Thus, to achieve a therapeutic effect, the drug can reach the epidermis and dermis in psoriatic lesions to interact with the immune cells and cytokines.

## Introduction

Psoriasis is a chronic autoimmune skin disease that affects 2–5% of the world’s population (Sala et al., 2016). The characteristic symptoms of psoriasis are erythema with sharp borders and covered with multiple layers of silvery white scales, which primarily occur on the scalp and extensor parts of the limbs (Krueger and Bowcock, 2005; Boehncke and Schön, 2015). Presently, the pathogenesis of psoriasis remains unclear, but it is believed to result from a combination of genetic, immunological, and environmental factors. Cross-contamination from unclean hair-cutting tools may contribute to the spread of the disease. Although psoriasis is a mild disease with localized inflammatory lesions, if an individual does not receive prompt and effective treatment, psoriasis can easily turn into a severe disease, with widespread plaques covering more than 10% of the body surface areas (Hawkes et al., 2017). Patients with psoriasis are at increased risk for psychological difficulties, including depression/anxiety and, in severe cases, suicidality (Dowlatshahi et al., 2014; Fleming et al., 2017; Singh et al., 2017).

The past decade has seen the rapid development of systematic studies on psoriasis, although there remains no treatment that can heal psoriasis. Generally, three types of treatments are provided, namely, local treatment, phototherapy, and systemic therapy. However, related toxicities cause poor compliance during long-term treatment (Rajitha et al., 2019), which restricts clinical adoption of these treatments. Hence, how to develop a new and effective therapy for psoriasis remains an urgent conundrum. Traditional medicine has been known and used in psoriasis for years, resulting in experience with therapies and prescriptions that provide the basis for the treatment of psoriasis. With deep research into traditional medicine through modern pharmacological and chemistry means, a large number of natural products (such as ferulic acid (Lo et al., 2019), curcumin (Kang et al., 2016), indirubin (Lin et al., 2014), and capsaicin (Kürkçüoğlu and Alaybeyi, 1990)) with anti-psoriasis activity have been unlocked. The drug administration of natural products can be commonly treated under five routes: intravenous injection (Zhou et al., 2017), intravenous drip (Shi et al., 2019), oral administration (Li et al., 2019a), phototherapy (Niu et al., 2015), and transdermal administration (Pal et al., 2015). Among them, transdermal drug delivery is the most commonly used alternative, allowing traditional dosage forms such as gels, creams, and tinctures to be utilized with natural products to treat psoriasis. However, transdermal drug delivery with natural products is limited by traditional formulations’ poor solubility and low permeability, which prevents the drug from being absorbed through the skin. The pathological and histochemical changes in psoriasis lead to exceptional skin permeability. On the one hand, the drugs have difficulty in reaching the epidermis through multiple layers of scales, reducing permeability. On the other hand, the recurrent shedding of scales due to the fragility of stratum corneum (SC) and the irregularity of corneocytes may increase permeability. It is now understood that ceramides play an essential role in the water retention of SC (Pinheiro et al., 2007; Coto et al., 2011; Liang et al., 2020). Therefore, the water holding capacity of skin affected by psoriasis is decreased because of the decline in ceramides (Sano, 2015). Consequently, novel drug delivery systems (such as liposomes, lipospheres, nanostructured lipid carriers, niosomes, and nanoemulsions) have been developed to improve the skin penetration of natural products, impacting the immune cells in the epidermis or dermis and therefore improving the therapeutic effects.

Here, we review recent studies on the development of conventional natural products and the novel drug delivery systems to enhance the skin drug delivery of natural products for the treatment of psoriasis.

## The Clinical Findings and Histopathological Features of Psoriasis

The clinical phenotypes of psoriasis are generally classified into the following types: plaque psoriasis, guttate psoriasis, pustular psoriasis, and erythrodermic psoriasis. Plaque psoriasis, accounting for 90% of all cases, is characterized by red skin with silvery scales, usually occurring in certain areas, including the knees, elbows, and scalp (Griffiths and Barker, 2007). The plaques vary in size, are symmetrically distributed, and are often surrounded by normal skin. The psoriatic skin and the normal skin are sharply demarcated from each other. Guttate psoriasis, whose typical features involve small and scattered papules, may be associated with streptococcal infections. Generalized pustular psoriasis, a relatively rare and severe form, manifests as superficial, sterile, and tender pustules on an erythematous base. Erythrodermic psoriasis, the most severe form of psoriasis, may be life threatening. The clinical expression of erythrodermic psoriasis involves bright erythema across the body and superficial desquamation.

The skin comprises the dermis and the epidermis, which has a multilayer structure comprising basal, spinous, and granular cell layers. During differentiation, keratinocytes can gradually move from the basal layer to differentiate into the upper layers of the epidermis and eventually desquamate (Madden et al., 2020b). The abnormal function of neutral lipids, which makes it difficult to secrete into the extracellular space, results in a defective water/vapor barrier and shedding of the SC. For normal skin, the time taken by keratinocytes to reach the surface of the skin from the basal layer is probably 6–8 days, whereas the time is probably 40 days in psoriatic skin. This phenomenon is presumably linked to the increased expression in keratin 6/16/17 in psoriasis lesions (Roberson and Bowcock, 2010). Histologically, there is a prominent hyperkeratosis, parakeratosis (retention of nuclei in cells of the SC), absence of the normal granular layer, and a thickened spinous layer. Additionally, psoriatic skin exhibits elongation of rete ridges that may develop into a long, fine, undulating morphology. Micro-abscesses of Munro, the characteristic hallmark of psoriasis, form because of the substantial number of neutrophils accumulating in the epidermis. Because of the thinning or disappearance of the granular layer at psoriasis lesions, the spinous layer may prematurely express the products (such as fatty acid-binding protein, filaggrin, corneodesmosin, glutaminase, involucrin, and loricrin) normally expressed in the granular layer (Ruissen et al., 1996; Guttman-Yassky et al., 2009). The blood vessels in the dermis are prominent and increase in number, which may relate to the epidermal vascular endothelial growth factor (VEGF) (Elias et al., 2008).

**FIGURE 1:**
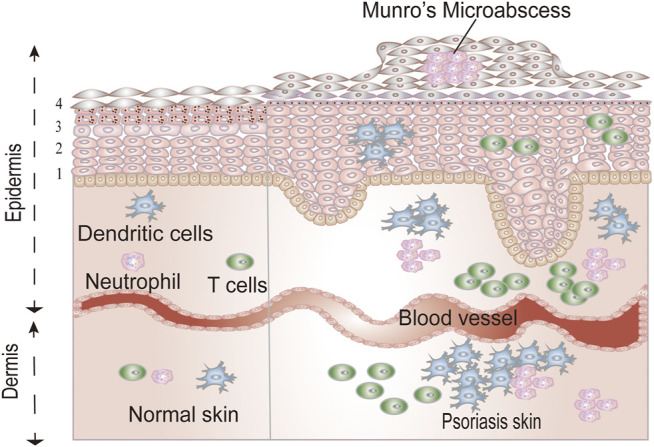
The differences between normal skin and psoriatic skin. The epidermis contains four homogenous layers: stratum basale (1), the stratum spinosum (2), the stratum granulosum (3), and the stratum corneum (4).

Other defining histological features of psoriasis are reflected by the infiltration of immune cells in the skin. In the dermis, there is marked infiltration of CD4^+^ T cells and dendritic cells (DCs), whereas in the epidermis, an exaggerated number of CD8+T cells, DCs, and neutrophils are found. Figure 1 illustrates the histological differences between psoriatic skin and normal skin.

## Pathogenesis of Psoriasis

Up to now, research into the pathogenesis of psoriasis has been exploratory. Before 1979, keratinocyte dysregulation, leading to keratinocyte hyperproliferation, was considered to contribute to the disease (Wagner et al., 2010; Lynde et al., 2014). In 1986, evidence emerged that psoriasis was associated with the activation of lymphocytes, infiltration of lymphocytes, and abnormal proliferation of keratinocytes (Valdimarsson et al., 1986). Presently, most investigators believe that the activation of immune cells (such as T cells, neutrophils, MCs, and macrophages), which secrete copious amounts of cytokines, eventually leads to the proliferation of keratinocytes, epidermal thickening, and angiogenesis.

### The Roles of DCs in Psoriasis

DCs, the professional antigen-presenting cells, comprised myeloid DCs (MDCs) and plasma cell-like DCs (pDCs). DCs are significant contributory factors to the development of psoriasis because of the cytokines secreted by them and their influence on T cells. MDCs are involved in immune regulation through the secretion of cytokines (such as interleukin [IL] 12, IL-23, tumor necrosis factor-alpha [TNF-α], IL-20, IL-8, and inducible nitric oxide synthase [iNOS]) (Johnson-Huang et al., 2012). IL-12 can induce the differentiation of naive T cells into Th1 cells, whereas IL-23 can induce them into Th17 and Th22 cells (Edmund et al., 2004; Zhou et al., 2007). The MDCs interact with T cells through the immunological synapse and stimulate T cells to secrete corresponding cytokines, thus causing a series of effects. Additionally, pDCs, which accumulate mainly in the blood and lymphoid tissues, are sensitive to the infection caused by a virus (Yong-Jun, 2005). Upon stimulation with the complexes of LL-37 and cell-free DNA (cfDNA), pDCs trigger toll-like receptor 9, leading to the differentiation and maturation of pDCs and the secretion of interferon (IFN)-α, which can promote the subtype of DCs from MDCs to pDCs and, thus, mediate the activation of T cells to secrete a series of cytokines. Moreover, IFN-α enhances the expression of IL-22R on epidermal keratinocytes, which is followed by the inhibition of keratinocyte differentiation, resulting in the thickening of the epidermis (Tohyama et al., 2012).

### The Roles of T Cells in Psoriasis

Over the last 2 decades, research has tended to focus on T cells, primarily including CD4^+^ T and CD8^+^ T cells, and their vital roles in the pathogenesis of psoriasis. Under the induction of MDCs or macrophages, naïve CD4^+^ T cells can differentiate into Th1, Th2, Th17, or Treg cells, which seem to work primarily by producing cytokines. For a long time, psoriasis was assumed to be mediated by Th1 cells through the secretion of TNF-α, IFN-γ, IL-2, and IL-12 (Austin et al., 1999). In 2007, Fitch and Kastelein et al. (Fitch et al., 2007; Kastelein et al., 2007) proposed an “IL-23/Th17 axis,” which might be more applicable in the present research of psoriasis. IL-23, mainly produced by activated DCs and macrophages, is the critical cytokine used to maintain the phenotype of Th17 cells (Stritesky et al., 2008). Th17 cells participate in immune regulation directly or via the production of IL-17, IL-22, and TNF-α. IL-22 can stimulate the signal transducers and activators of transcription (STAT) three signaling pathway and then stimulate proliferation and differentiation of keratinocytes, and thus inducing keratinocytes to produce chemokines (such as CXCL8 and CXCL1) (Sestito et al., 2011). IL-17 has been found to promote the proliferation of keratinocytes and the production of cytokines (IL-1β, IL-6, and TNF) and antimicrobial peptide (β-defensin and matrix metalloproteinase). Furthermore, chemokines (CXCL8 and CXCL1) produced by IL-17 may, in turn, result in the recruitment of Th17 and DCs in the epidermis, thus forming a positive feedback loop (Nograles et al., 2008; Girolomoni et al., 2017). Th22 cells act primarily through the secretion of IL-22 and TNF-α.

**FIGURE 2:**
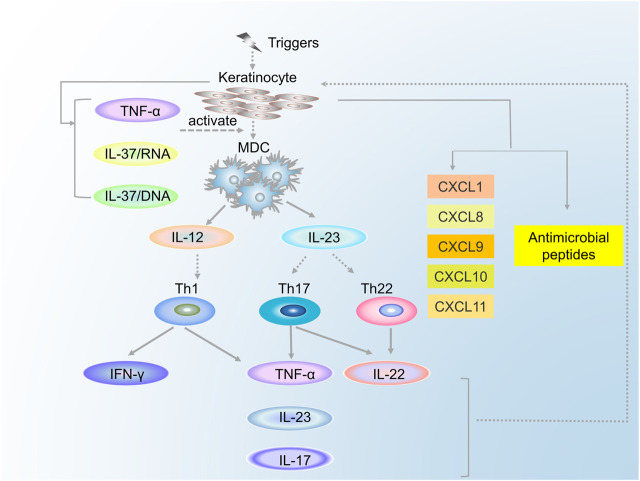
The relationship between the MDCs and the T cells.

Similar to CD4^+^ T cells, there also exist T-cell subsets in the CD8^+^ cells, which can secrete cytokines such as IL-2, IL-17, and IL-22: these cytokines were named Tc1, Tc17, and Tc22, respectively. Tc1 cells secrete IFN-γ, IL-2, and TNF-α, whereas Tc22 cells function by secreting IL-22. Compared with Th17 cells, Tc17 cells secrete not only IL-17, IL-22, TNF-α, and IFN-γ but also CCL20 and Granzyme B. IL-17A is key both in creating keratinocyte hyperproliferation and in producing other proinflammatory cytokines (Volarić et al., 2019). Although there have been relatively few studies of CD8^+^ cells in psoriasis, it is now clear that these cells can produce corresponding cytokines, which can cause the pathologic changes in keratinocytes and blood vessels. The relationship between the MDCs and the T cells is shown in Figure 2.

### The Roles of Neutrophils in Psoriasis

Neutrophils and their release of genomic, DNA-based, web-like structures known as neutrophil extracellular traps (NETs) play an essential role in the regulation of psoriasis. Neutrophils, the most abundant leukocytes in the human peripheral vascular system, can be drawn to the epidermis of psoriasis lesions in large numbers. In psoriasis lesions, the CXCR2 ligand released by keratinocytes could promote neutrophil migration from blood vessels to the epidermis and cooperatively increase the neutrophil accumulation in combination with Leukotriene B4. The IL-1β produced by neutrophils stimulates keratinocytes to secrete IL-1, IL-19, and CXCR2 ligands, which in turn act on neutrophils, thereby forming an immune circulation system (Sumida et al., 2014). Additionally, the matrix metallopeptidase-9 overexpressed by neutrophils can activate vascular endothelial cells, which may lead to skin vascular expansion and enhance permeability (Chen et al., 2020).

Recently presented data indicate that DCs are activated by the LL-37 and cfDNA complexes, thus producing a sequence of immune responses leading to psoriasis. Conversely, NETs might be the source of LL-37 and cfDNA complexes that accumulate in the psoriatic skin. When LL-37 binds to RNA (DNA), it may stimulate neutrophils to release LL-37 and various cytokines, thus forming a self-propagating, vicious cycle (Herster et al., 2020). Additionally, NETs might interact with T cells to impact the immune response to psoriasis. For example, NETs could enhance the expression of Act1 and D10N (key mediators of IL-17 signal transduction), which can lead to the production of Th17 in the presence of a variety of monocytes (Lambert et al., 2019).

### Therapies to Treat Psoriasis

To date, the available treatments cannot completely cure psoriasis, and existing therapies only mitigate the symptoms to improve patients’ quality of life. Treatment options for the management of psoriasis generally include local treatment, phototherapies, and systemic treatments, which are utilized according to the different characteristics of the psoriasis.

Depending on the severity of symptoms, psoriasis can be classified as mild, moderate, or severe. Local treatment, mainly including corticosteroids and vitamin D and its analogs, is often the first choice for patients with mild to moderate symptoms (Pariser et al., 2007). However, phototherapy and systemic treatments should be considered if local treatment does not achieve the desired results or the disease becomes more serious. Phototherapies, based on broad-spectrum ultraviolet B and narrow-spectrum ultraviolet A (UVA), are used in combination with local treatment to improve efficacy. However, phototherapy treatments should not be used in patients who have hypersensitivity to light, cataracts, or liver/kidney failure. Systemic treatment, typically including oral retinoids (acitretin), cyclosporine, or methotrexate, should be considered when both local treatment and phototherapy fail to work. Long-term use of a systemic treatment may lead to adverse reactions, such as hypertension, nephrotoxicity, hepatotoxicity, and hyperlipidemia. Natural products possess the advantages of high efficiency and low toxicity and thus are highly promising therapy options for psoriasis.

## The Conventional Natural Products for the Treatment of Psoriasis

### Flavonoids

Flavonoids, a kind of polyphenolic compounds in plants, belong to the secondary metabolites of plants. Flavonoids have been shown to possess medicinal properties, and their remarkable anti-inflammatory effect plays an important role in the treatment of psoriasis.

To evaluate the anti-psoriasis effect of aromatic-turmeron, Li et al. (Li et al., 2018b) treated IMQ-induced mice topically with aromatic-turmeron at 40 mg/kg (high dose) or 0.4 mg/kg (low dose). The results showed that both doses of aromatic-turmeron reduced the PASI score of psoriatic mice and the thickness of the epidermis. Meanwhile, compared with that in the model group, the content of CD8^+^ T cells in the epidermis was significantly reduced in the treatment group, with approximately one-fifth of the CD8^+^ T cells reduced in the low-dose treatment group and nearly half reduced in the high-dose treatment group. The expression of TNF-α and IL-6 was decreased in the treatment group, and the messenger RNA (mRNA) synthesis of IL-17, IL-22, and IL-23 was downregulated. Notably, the high-dose treatment group reduced the expression level of IL-6 to similar levels as the control group. Therefore, aromatic-turmeron is a candidate drug for the treatment of psoriasis.

Additionally, Lv et al. (Lv et al., 2020) investigated the efficacy and mechanisms of luteolin in treating psoriasis. The *in vitro* results showed that luteolin inhibited the transcription expression of Hsp90 and the secretion of Hsp90 in HaCaT cells. The *in vivo* study demonstrated that the PASI scores of IMQ-induced mice were reduced to nearly 1, and the inflammatory cells at the lesion were reduced after intraperitoneal injection with luteolin on the eighth day. Luteolin was found to inhibit the exosome secretion of Hsp90 in the plasma of the psoriasis mice. Therefore, luteolin can regulate the expression level and exosome secretion of Hsp90 to achieve therapeutic effects. Moreover, It is hypothesized that the skin protective effects of luteolin could derive by the putative interactions with tyrosinase, whose inhibition has been related to the treatment of psoriasis (Chai et al., 2017; Nagula and Wairkar, 2019; Alalaiwe et al., 2020). Table 1 shows other flavonoids for the treatment of psoriasis.

**TABLE 1:** Flavonoids for the treatment of psoriasis.

Natural compound	Experimental model	Therapy	Dose	Dosage form	Mechanism of action	References
Amentoflavone	IMQ-induced BALB/c mice/M5 (a cocktail of cytokines)-treated HaCaT cells	Oral administration	25 mg/kg	—	Inhibited the increase of expression of cyclin D1, cyclin E, IL-17A, and IL-22, suppressed the expression of NF-κB	An et al. (2016)
Epigallocatechin-3-gallate	IMQ-induced BALB/c mice	Topical application	—	Solution (glycerin solvent: 50% glycerin, 50% normal saline)	Reduced the expression of epidermal PCNA, promoted the expression of caspase-14	Zhang et al. (2016)
Cyanidin	Primary human keratinocytes	—	20 μM	—	Upregulated expression of all five LCE3 genes	Austin et al. (2014)
Astilbin	IMQ-induced BALB/c mice	Oral administration	50 mg/kg	Solution (dissolved in DMSO, then diluted in normal saline)	Ameliorated elevations in circulating CD4^+^ and CD8^+^ T cells and inflammatory cytokines (IL-17A, TNF-α, IL-6, IFN-γ, and IL-2)	Di et al. (2016)
Quercitrin	IMQ-induced C57BL/6 mice	Oral administration	50 mg/kg	Solution (dissolved vehicle [Ethanol: Normal saline: Tween-80: Peanut oil = 12:23:5:60, V/V])	Lowered the expression of cytokines related to psoriasis, especially those on the IL-23/Th17 axis, inhibited the Th17 cell response regulated by the JAK/STAT signaling pathway	Chen et al. (2017c)
Quercetin	IMQ-induced BALB/c mice	Oral administration	120 mg/kg	Suspensions (resuspended in 0.5% carboxy-methyl cellulose sodium)	Downregulated the expression of NF-κB, IKKα, NIK, and RelB and up-regulated the expression of TRAF3	Chen et al. (2017a)
Naringenin	IMQ-induced BALB/c/IMQ-stimulated keratinocytes	Topical application	—	—	Suppressed IL-6 over expression to baseline control	Alalaiwe et al. (2020)
Glabridin	IMQ-induced BALB/c mice/HaCaT cells	Topical application	10 mg/kg	Solution (dissolved in butanediol)	Inhibited the expression of IL-6, IL-1β, IL-17A, TNF-α, IL-22, and IL-23 and CCL2	Li et al. (2018a)
Hesperidin	IMQ-induced BALB/c mice/LPS-stimulated HaCaT cells	Oral administration	125 mg/kg	Solution (dissolved in pure water)	Modulated the secretion levels of serum leptin, adiponectin, and resistin and inhibited the activation of the IRS-1/erk1/2	Li et al. (2019a)
Hispidulin	IMQ-induced C57BL/6 J mice/Activated keratinocytes	Oral administration	0.1 mg/kg	—	Alleviated pathologically increased levels of immunoglobulin G2a, myeloperoxidase, and TNF-α, splenic, reduced Th1 and Th17 cell populations, and inhibited gene expression of Th1- and Th17-associated cytokines and chemokines, and phosphorylation of mitogen-activated protein kinases and NF-κB	Kim et al. (2020)
Luteolin	TNF-triggered human keratinocytes	—	10 μM	Solution (dissolved in DMSO)	Inhibited the production of inflammatory mediators IL-6, IL-8, and VEGF	Weng et al. (2014)
Taxifolin	IMQ-induced BALB/c mice/LPS-induced HaCaT cells	Oral administration	40 mg/kg	Solution (dissolved in distilled water)	Inhibited Notch1 and JAK2/STAT3 signal pathways	Yuan et al. (2020)
Proanthocyanidin	IMQ-induced BALB/c mice	Topical application	200 mg/kg	—	Decreased inflammatory cell infiltration and downregulated expression of the psoriasis-associated genes IL17a, IL22, S100a9, and Krt1 and inhibited arachidonate 5-lipoxygenase	Toda et al. (2020)
Delphinidin	Normal human epidermal keratinocytes (NHEKs)	—	10–40 μM	Solution (dissolved in DMSO)	Enhanced cornification and increased the protein expression of cornification markers, including caspase-14 and keratin 1	Chamcheu et al. (2013)
Delphinidin	Full-thickness three-dimensional reconstituted human skin	—	20 μM	Solution (dissolved in DMSO)	Induced the mRNA and protein expression of markers of differentiation (caspase-14, filaggrin, loricrin, and involucrin)	Chamcheu et al. (2015)
Delphinidin	Flaky skin mice (fsn/fsn)	Topical application	0.5 mg/cm^2^	—	Reduced pathological markers of psoriasiform lesions, infiltration of inflammatory cells, and the mRNA and protein expression of inflammatory cytokines	Pal et al. (2015)
Rhododendrin	IMQ-induced C57BL/6 mice/normal human epidermal keratinocytes	Topical application	20 mM	—	Reduced inflammatory mononuclear cell infiltration and the expression of pro-inflammatory mediators and inhibited the activation of the TLR-7/NF-κB and mitogen-activated protein kinase pathways	Jeon et al. (2017)
Baicalein	HaCaT keratinocytes	—	10 μM	—	Increased expression of keratins 1 and 10 (K1/K10) and increased the phosphorylation of ERK, AKt, and p38 MAPK.	Huang et al. (2016)
Genistein	HaCaT cells	—	—	Solution (dissolved in DMSO)	Suppressed ROS activation and reduced the RNA and protein level of cytokine (IL-8, IL-20, and CCL2)	Amodio et al. (2018)

### Phenylpropanoids

Phenylpropanoids, including coumarins, phenylpropionic acids, curcumin, and gallic acid, showed promising results for the treatment of psoriasis.

#### Coumarins

Coumarins, an essential class of natural products, possess a benzoquinone α-pyran one core (Garg et al., 2020). Coumarins have been found to reduce the expression of inflammatory cytokines and chemokines, thereby exerting anti-psoriatic action. Typically, coumarins, represented by psoralen, are combined with phototherapy to improve therapeutic effects in psoriasis. Carrascosa et al. (Carrascosa et al., 2013) investigated the effectiveness and safety of psoralen-UVA (PUVA) therapy for 48 patients with a mean age of 51 years with palmoplantar psoriasis. After topical PUVA therapy, 63% of the cases were considered to be effectively treated (PGA score of 0 or 1). However, topical PUVA therapy should be combined with acitretin in cases where PUVA therapy has no apparent effect after 8 to 10 sessions. During treatment, 25% of the patients reported adverse effects. Overall, PUVA therapy achieved better results and had a favorable safety profile.

#### Phenylpropionic Acids

Phenylpropionic acids, such as ferulic acid and danshensu, have demonstrated favorable results in psoriasis. Lo et al. (Lo et al., 2019) aimed to study the therapeutic effects of ferulic acid on psoriasis by using psoriasis mice orally administered with ferulic acid (100 mg/kg) for 14 consecutive days. The skin lesions of the mice were significantly ameliorated at Day 8 after the ferulic acid treatment. Compared with the model group, the scaling score and erythema score were reduced by about 30 and 70%, respectively. Ferulic acid decreased the gene expression of IL-17A and prevented IL-17A from binding to IL-17RA.

Danshensu is a traditional Chinese herbal medicine used for the treatment of psoriasis, but its underlying mechanisms remain unclear (May et al., 2015). Jia et al. investigated the anti-psoriasis impacts of danshensu on psoriasis mice after intraperitoneal injections (20, 40, and 80 mg/kg/day) given on seven consecutive days. Danshensu improved the skin scales and thicknesses of the psoriasis in a dose-dependent manner. Compared with that in the model group, the skin thickness was decreased by 80% after high-dose treatment. In the high-dose treatment group, the expression of the YAP protein in the skin tissues was reduced to the level of the control group. Danshensu may achieve its anti-psoriatic effects by inhibiting the expression of YAP.

#### Other Phenylpropanoids

Curcumin, a polyphenolic compound extracted from the roots of curcuma longa, has unique advantages for the treatment of psoriasis. Kang et al. (Kang et al., 2016) evaluated the anti-psoriasis effects of curcumin through treating keratin 14-VEGF transgenic mice orally (40 mg/kg). The Baker scores of the mice were remarkably reduced after 10–20 days of treatment with curcumin. Compared with the model group, the Baker scores were significantly reduced by approximately 60% after 10 days of curcumin treatment. After 20 days of treatment, curcumin could reduce the levels of TNF-α, IFN-γ, IL-2, IL-12, IL-22, and IL-23 in the psoriasis mice to nearly 40–50%. After an additional 20 days, T cells in the ear skin could be reduced by curcumin treatment. More importantly, the results showed that there were no side effects for the kidney.

Gallic acid is a natural, small molecule, which shows a wide spectrum of important pharmacological properties, among which an anti-psoriasis effect has been the focus of research attention in recent years. Zhang et al. (Zhang et al., 2018) used gallic acid to treat psoriasis mice for six consecutive days. After 4 days of treatment, gallic acid significantly reduced the PASI scores of psoriatic mice. Meanwhile, compared with the model group, the gallic acid treatment group could reduce the thickness of the epidermis by more than 50% and reduce the dermal infiltrating cells. Gallic acid decreased the mRNA and protein expression of keratin 16 and keratin 17 and downregulated the activity of Nrf2 in mice with psoriasis. Table 2 shows other phenylpropanoids for the treatment of psoriasis.

**TABLE 2:** Phenylpropanoids for the treatment of psoriasis.

Natural compound	Experimental model	Therapy	Dose	Dosage form	Mechanism of action	References
Isopsoralen	IMQ-induced BALB/c mice	Topical application	0.03 mg/cm^2^	Solution (dissolved in PEG400/pH7.4 buffer (1:4)	Downregulated the expression of IL-6	Alalaiwe et al. (2018)
Psoralen	Patients with plaque-type psoriasis	Oral administration	25 mg/m^2^ (based on body surface area)	—	—	de Berker et al. (1997)
Psoralen	Patients (psoriasis area and severity index score of 8–15)	Topical application	—	Cream	—	Grundmann-Kollmann et al. (2004)
8-Methoxypsoralen	Inpatients with psoriasis vulgaffs	Oral administration	0.6 mg/kg	—	Suppressed the migrational activity of circulating neutrophils and monocytes and reduced the chemotactic activity in the *epidermis*	Ternowitz. (1987)
8-Methoxypsoralen	K5.hTGF-β1 transgenic mice	Topical application	—	Tincture	Suppressed the IL-23/Th17 pathway, Th1 milieu, transcription factors STAT3 and orphan nuclear receptor RORgt and induced the Th2 pathway and IL-10–producing CD4^+^ CD25 ^+^ Foxp3 + tregs	Singh et al. (2010)
Curcumin	HaCaT cells	Phototherapy	0.2–1 μg/ml	Solution (dissolved in DMSO)	Increased fragmented cell nuclei, the release of cytochrome c from mitochondria, activated caspase-9 and caspase-8, inhibited NF-κB activity, and inhibited extracellular regulated kinases 1/2 and protein kinase B	Dujic et al. (2007)
Curcumin	HaCaT cells	Phototherapy	1.25–3.12 μM	Solution (dissolved in DMSO)	Inhibited NF-κB activity and activated caspase-8 and caspase-9 while preserving the cell membrane integrity and downregulated the phosphorylation level of Akt and ERK	Niu et al. (2015)
Curcumin	IMQ-induced BALB/c mice	Topical application	50 mg/cm^2^	Gel (curcumin hydroxypropylcellulose gel)	Decreased the mRNA levels of IL-17A, IL-17F, IL-22, IL-1β, IL-6, and TNF-α and downregulated IL-17A/IL-22 production	Tanowitz et al. (2013)
Curcumin	HaCaT cells	—	50 μM	—	Downregulated the expression of IL-17, IL-6, TNF-α, and IFN-γ and up-regulated the expression of involucrin and filaggrin	Varma et al. (2017)
Curcumin	TNF-α-treated HaCaT cells	—	7.37 μg/ml	Solution (dissolved in 50% propylene glycol PBS solution)	Upregulated the expression of TRAIL-R1/R2 and inhibited the TNF-α–induced production of IL-6/IL-8 in HaCaT cells	Sun et al. (2012)
Resveratrol	IMQ-induced BALB/c/AnNTac mice	Oral administration	400 mg/kg	—	Increased the expression of RXR and decreased the expression of IL-17 dependent pathways, IL-17A, IL-17F, and IL-19	Bobé et al. (2015)
Resveratrol	HaCaT cells	—	50 μM	—	Inhibited the Akt pathways by inducing Sirt1	Lee et al. (2016)
Resveratrol	Normal human epidermal keratinocyte	—	40 μM	—	Downregulated the expression of AQP3 via an SIRT1/ARNT/ERK-dependent manner	Wu et al. (2014)

### Terpenoids

Terpenoids, a group of hydrocarbons biosynthetically derived from isoprene units, have a wide range of biological activities, such as antibacterial, anti-neurotoxicity, and antiviral immunosuppression. Moreover, terpenoids, as a kind of natural product with anti-inflammatory multi-target activity, have strong potential for the treatment of psoriasis. Liu et al. (Liu et al., 2019) investigated the anti-psoriasis effects of betulinic acid on psoriasis mice. Compared with the model group, the PASI scores of the betulinic acid treatment group were significantly reduced by nearly 40% and had an epidermis that was nearly 60% less thick. The infiltration of CD3^+^ T cells in the skin was significantly reduced by betulinic acid treatment. Betulinic acid suppressed Th17, γδT development, and NF-κB signaling.

Although its mechanism remains unknown, paeoniflorin is clinically efficacious in the treatment of psoriasis. Sun et al. (Sun et al., 2015) investigated the therapeutic effects and mechanism of paeoniflorin on IMQ-induced psoriasis mice. After 16 days of intraperitoneal paeoniflorin treatment (150 mg/kg), the ear thickness of psoriatic mice was significantly reduced. The histopathology assay showed that paeoniflorin treatment ameliorated the skin inflammation and reduced the epidermis thickening. The number of F4/80^+^ CD68 ^+^ macrophages and CD11b^+^ Gr-1^+^ neutrophils in the skin was decreased by paeoniflorin application. Paeoniflorin reduced the levels of IL-1β, IL-6, IL-12, IL-23, TNF-α, and iNOS. Table 3 shows other terpenoids for the treatment of psoriasis.

**TABLE 3:** Terpenoids for the treatment of psoriasis.

Natural compound	Experimental model	Therapy	Dose	Dosage form	Mechanism of action	References
Andrographolide	IMQ-induced C57/BL6 mice	Oral administration	5 mg/kg	—	Reduced expression of IL-23 and IL-1β in the skin	Shao et al. (2016)
Artesunate	IMQ-induced BALB/c mice	Intraperitoneally injected	60 mg/kg	—	Inhibited the expression of γδ T cells in draining lymph nodes	Huang et al. (2019)
Cycloastragenol	IMQ-induced C57BL/6 mice	Oral administration	25 mg/kg	—	Selectively modulated macrophage function by inhibiting NLRP3 inflammasome-mediated pyroptosis	Deng et al. (2019)
3β,6β,16β-trihydroxylup-20 (29)-ene	12-O-tetradecanoylphorbol-acetate induced swiss mice/HaCaT cells	Topical application	0.656 μmol/ear	Solution (dissolved in 20 μL of ethanol-acetone (1:1))	Activated corticosteroid receptors	Horinouchi et al. (2017)
Celastrol	Human HaCaT keratinocytes	—	2.2 μM	Solution (dissolved in DMSO)	Inhibited the NF-κB activity	Zhou et al. (2011)
Dehydrocostuslactone and costunolide	Human keratinocytes	—	12.5 μM	—	Counteracted the proinflammatory effects of IFN-γ and IL-22 on keratinocytes and reverted the apoptosis-resistant phenotype	Bachmann et al. (2014)
Paeoniflorin	IMQ-induced C57BL/6 mice	Oral administration	120 mg/kg	Solution (dissolved in normal saline)	Regulated Th17 cell response and cytokine secretion via phosphorylation of STAT3	Zhao et al. (2016)

### Alkaloids

Alkaloids, a class of basic compounds containing nitrogen, are ubiquitously distributed in plants, bacteria, and fungi. Alkaloids, including indirubin, oxymatrine, and capsaicin, are considered to have the potential for the treatment of psoriasis because of their strong anti-inflammatory activities.

Indirubin is the main active substance of indigo naturalis, which is commonly applied to treat psoriasis in clinics (Lin et al., 2017). In recent years, research into the underlying mechanisms of indirubin on psoriasis has mainly focused on its anti-proliferative effect on keratinocytes and the inflammatory response mediated by immune cells. Studies have shown that indirubin exerts anti-psoriatic action by inhibiting the activation of EGFR and the gene expression of EGF-induced CDC25B in epidermal keratinocytes (Hsieh et al., 2012). Xie et al. (Xie et al., 2018) investigated the therapeutic effect of indirubin on psoriasis. On Day 7, the PASI scores in the indirubin treatment group (50 mg/kg, orally administered) were reduced by almost half compared with those in the model group. Indirubin also reduced the infiltration of CD3^+^ T cells, CD11b^+^ neutrophils, and IL-17A–producing γδT cells, thereby improving keratinocyte proliferation. Furthermore, indirubin inhibited the mRNA expression of IL-1, IL-6, IL-17a, IL-22, and IL-23.

Oxymatrine, an active component of *Sophora flavescens*, showed excellent anti-inflammatory and anti-psoriatic effects. To investigate the potential of oxymatrine in the treatment of psoriasis, Chen et al. (Chen et al., 2017b) gathered 150 patients clinically diagnosed with psoriasis vulgaris from both inpatient and outpatient clinics. They were given 600 mg/kg oxymatrine intravenously for 4 weeks. The efficacy rate of oxymatrine was 78% and the PASI scores of the patients decreased from 11.66 to 2.91 after oxymatrine treatment. During the treatment, there were no systemic adverse events.

Capsaicin, a major biologically active component of chili peppers, is commonly applied topically to treat psoriasis (Arnold and van de Kerkhof, 1994). Evidence has emerged showing that capsaicin can effectively treat psoriasis. Ellis et al. (Ellis et al., 1993) conducted a clinical trial with a double-blind, vehicle-controlled, parallel-group, and multicenter design to investigate the therapeutic effects of topical application of capsaicin. Compared with vehicle-treated patients, patients who applied capsaicin 0.025% cream showed better pruritus relief, which may be related to the decrease of substance-P (SP). This view that the therapeutic effect of capsaicin is associated with the reduction of SP expression is also supported by a study from Kürkçüoğlu et al. (Kürkçüoğlu and Alaybeyi, 1990). Table 4 shows other alkaloids used to treat psoriasis.

**TABLE 4:** Alkaloids for the treatment of psoriasis.

Natural compound	Experimental model	Therapy	Dose	Dosage form	Mechanism of action	References
Indirubin	IMQ induced BALB/c mice/Primary human epidermal keratinocytes	Subcutaneously given	50 mg/kg	—	Increased the level of CD274 in epidermal keratinocytes and alleviated the symptom of psoriasis-like mice depending on CD274	Xue et al. (2018)
Cannabinoids	Keratinocytes	—	—	—	Inhibited keratinocyte proliferation	Wilkinson and Williamson. (2007)
Rutaecarpine	IMQ induced BALB/c mice	Topical application	—	Cream	Inhibited the NF-κB and TLR-7 pathways	Li et al. (2019b)
Isocamptothecin	HaCaT cells	—	51 μg/ml	—	Downregulated the telomerase activity of HaCaT cells	Lin et al. (2008)
Acridones	HaCaT keratinocytes	—	—	—	Inhibited human keratinocyte growth	Putic et al. (2010)
Phytosphingosine derivatives	IL-23-induced C57BL/6 mouse	Topical application	—	—	Inhibited NF-κB, JAK/STAT, and MAPK signaling	Kim et al. (2014)
Oxymatrine	Patients with psoriasis	Intravenous drip	0.6 g daily	Solution	Inhibited the proliferation of epidermal cells in the skin lesion and increased Bcl-2 expression and decreased TUNEL-positive cells	Shi et al. (2019)
Oxymatrine	IMQ induced C57BL/6 mice/HaCaTs cells	Intraperitoneal injected	15 mg/kg	Solution (dissolved in sterile saline)	Inhibited the expression of Hsp90 and Hsp60 in keratinocytes through the MAPK signaling pathway	Xiang et al. (2020)
Oxymatrine	Patients with severe plaque psoriasis	Intravenous	0.6 g daily	Solution	—	Zhou et al. (2017)

### Steroids

Steroids, an essential group of natural products, are characterized by the presence of a cyclopentano-perhydrophenanthrene carbon skeleton in the molecular parent structure. Steroids exert a broad range of pharmacological properties, especially in the treatment of psoriasis. Wu et al. (Wu et al., 2020) examined the potential of diosgenin in the treatment of psoriasis. The PASI scores were significantly improved on Day 4 of treatment. By Day 7 of treatment, the PASI score was five in the treatment group, which is half of the score observed in the model group. Diosgenin downregulated proinflammatory cytokines and upregulated the expression of several differentiation markers.

### Organic Acids

Organic acids, which possess excellent therapeutic effects for psoriasis, are acidic organic compounds containing carboxyl groups, such as salicylic acid and gambogic acid. Salicylic acid, a clinical anti-psoriasis drug, is usually applied topically in combination with tacrolimus, mometasone furoate, or calcitriol to reduce side effects and increase the permeability of the drug to the skin. Salicylic acid hydrates and softens psoriasis skin by dissolving the intercellular cement or reducing the pH value of the SC (Lebwohl, 1999). In 408 patients with psoriasis, Koo et al. (Koo et al., 1998) treated one group with the combination of salicylic acid 5% and ointment mometasone furoate 0.1% and one group with mometasone furoate 0.1% ointment alone. The results showed that the combination of mometasone furoate and salicylic acid was more effective and safer than mometasone furoate alone. However, salicylic acid has certain toxic side effects, including skin irritation, liver injury, nausea, and vomiting. Therefore, the dosage and the application method of salicylic acid should be considered carefully.

**FIGURE 3:**
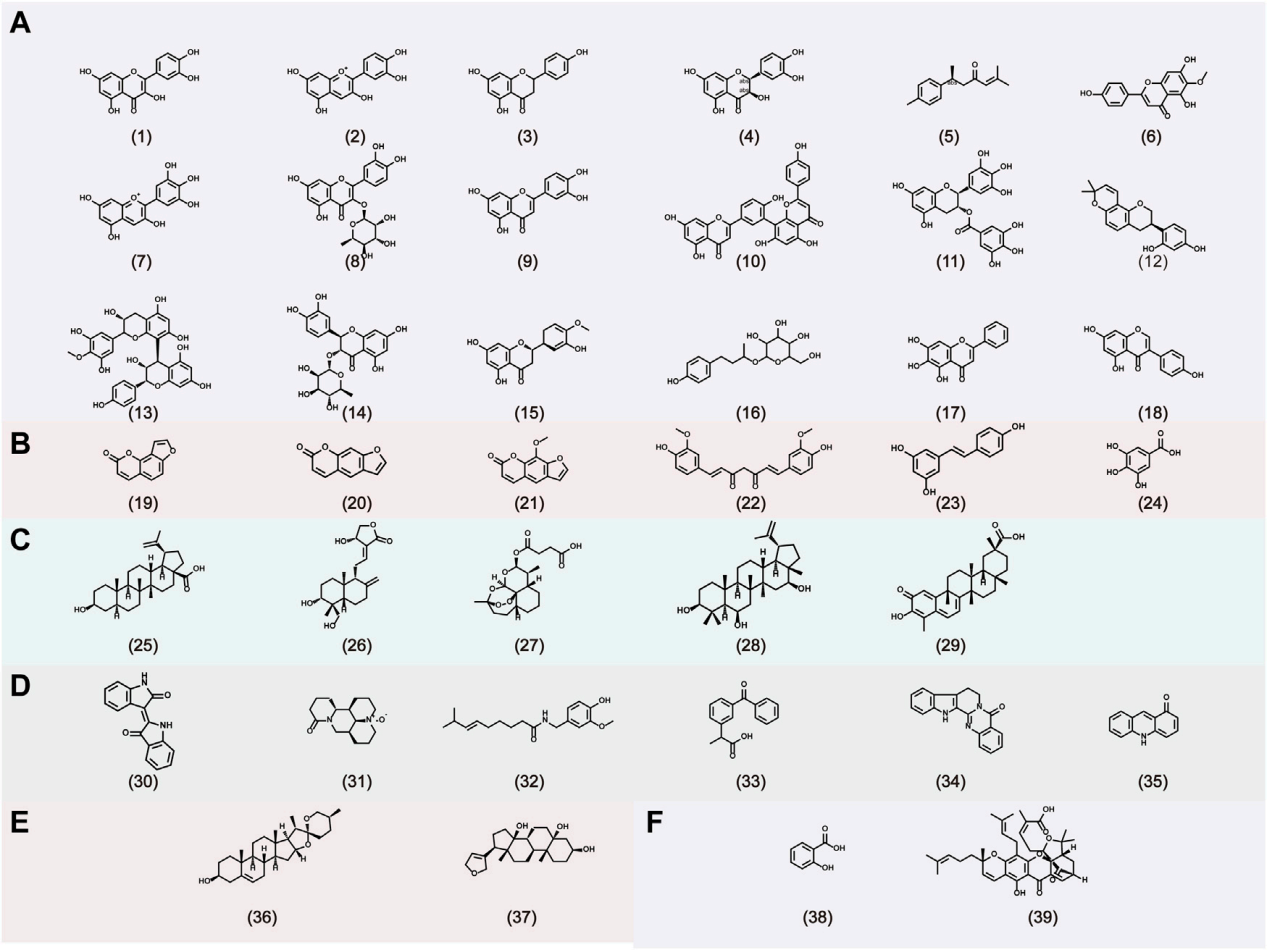
The chemical structures of natural products for the treatment of psoriasis. A: Flavonoids, B: Phenylpropanoids, C: Terpenoids, D: Alkaloids, E: Steroids, F: Organic acids. (1) Quercetin, (2) Cyanidin, (3) Naringenin, (4) Taxifolin, (5) Aromatic-turmerone, (6) Hispidulin, (7) Delphinidin, (8) Quercitrin, (9) Luteolin, (10) Amentoflavone, (11) Epigallocatechin-3-gallate, (12) Glabridin, (13) Proanthocyanidin, (14) Astilbin, (15) Hesperetin, (16) Rhododendrin, (17) Baicalein, (18) Genistein, (19) Isopsoralen, (20) Psoralen, (21) 8-methoxypsoralen, (22) Curcumin, (23) Resveratrol, (24) gallic acid, (25) Betulinic acid, (26) Andrographolide, (27) artesunate, (28) 3β,6β,16β-Trihydroxylup-20. (29)-ene (29) Celastrol, (30) Indirubin, (31) Oxymatrine, (32) Capsaicin, (33) Cannabinoids, (34) Rutaecarpine, (35) Acridones, (36) Diosgenin, (37) Periplogenin, (38) Salicylic acid, (39) Gambogic acid.

Gambogic acid, the main active compound isolated from the resin of the tree *Garcinia hanburyi*, has been recently reported to exhibit anti-psoriatic efficacy. Wen et al. (Wen et al., 2014) investigated the anti-psoriatic effects of gambogic acid. On gross inspection, the lesions, epidermal architecture, and the parakeratosis of K14-VEGF transgenic mice were improved after treatment with gambogic acid for 2 weeks. The Baker scores in the second, fourth, and sixth weeks were 2.6, 1.3, and 1.1, respectively, showing a time-dependent effect. Additionally, gambogic acid suppressed hyperplastic and inflamed vessels, decreased the expression of adhesion molecules (such as ICAM-1 and E-selectin), and reduced the expression of IL-17 and IL-22. The Chemical structures of natural products for the treatment of psoriasis is shown in Figure 3.

## Anti-psoriasis Effects of Natural Products Based Novel Drug Delivery Systems

In psoriatic lesions, the decreased amounts of ceramides in the skin and the impaired skin barrier function contribute to the abnormal permeability and the poor hydration capacity. Hence, effective drugs for psoriasis should exhibit desirable permeability and a good water holding capacity. Substantial therapeutic efficacy for psoriasis was exhibited by topical dosage forms of natural products for psoriasis, including gels, ointments, tinctures, emulsions, and liniments. Despite the great therapeutic efficiency of natural products, their application is limited by the poor permeability and lack of targeted enrichment into the dermis and epidermis. Therefore, novel drug delivery systems should be developed to enhance the skin permeability and targeting function of natural products and, consequently, to improve their therapeutic effects.

Overall, the following three conditions for psoriasis need to be satisfied. 1) The basic carrier should possess hydration power. 2) Novel drug delivery systems, such as liposomes, liposphere nanostructured lipid carriers, niosomes, nanoemulsions, nanospheres, microneedles, ethosomes, nanocrystals, and foams, can effectively improve the permeability of drugs and increase drug content in skin, thus interacting with inflammatory cells to improve efficacy. 3) The novel drug delivery systems should exhibit some targeting ability toward the dermis, epidermis, or hair follicle to interact with specific inflammatory cells or cytokines; this approach will limit the amount of the drug that goes into the systemic circulation and maximize the amount locally concentrated on the skin. The advantages/disadvantages of nanoformulation for the treatment of psoriasis are shown in Table 5.

**TABLE 5:** Nanoformulation-based natural products for the treatment of psoriasis.

Nanoformulation	Category	Drug	Drug limitations	Preparation method	Therapy	Remark	References
Liposomes	Alkaloids	Capsaicin	Irritating, burning effects on the skin	The thin-film hydration method	Topical application	Reduced the side effects of the drug and increased the therapeutic efficacy	Gupta et al. (2016)
Ethosomes	Phenylpropanoids	Psoralen	Poor permeability	The injection ultrasonic combination method	Topical application	Enhanced cutaneous absorption of drugs and permeability	Zhang et al. (2014b)
Niosomes	Phenylpropanoids	8-Methoxypsoralen	Gastrointestinal adverse effects and a higher risk of severe complications	The thin film hydration method	Psoralen ultraviolet a (PUVA) therapy	Promoted penetration and accumulation of 8-Methoxypsoralen	Kassem et al. (2017)
NLCs	Alkaloids	Pentoxifylline	Poor permeability	The thin lipid film-based microwave-assisted rapid technique	Topical application	Enhanced anti-inflammatory potential	Ghate et al. (2019)
	Phenylpropanoids	Curcumin	Highly hydrophobic molecule with low water solubility	The hot emulsification method	Topical application	Improved permeation and skin retention, showed no toxicity toward keratinocyte cells	Rapalli et al. (2020)
	Terpenoids	Thymol	High volatility and can also decompose due to heat, humidity, oxygen, or light	The sonication method	Topical application	Enhanced anti-inflammatory potential	Pivetta et al. (2018)
	Terpenoids	Tripterine	Long-term oral administration of TRI can lead to toxicity on renal and reproductive systems	The emulsification evaporation method	Topical application	Revealed the sustained release characteristics, showed great stability and biocompatibility	Kang et al. (2019)
Nanoemulsions	Terpenoids	Paclitaxel	Poor solubility and permeability	Low energy emulsification methods	Dermal/Oral delivery	Enhanced absolute and per oral bioavailability	—
	Phenylpropanoids	8-Methoxypsoralen	Poor permeability	High-energy method	Topical application	Enhanced retention in viable skin	Oliveira et al. (2018)
	Organic acids	Salicylic acid	Poor aqueous solubility and instability	Low-energy, spontaneous emulsification method	Topical application	Improved anti-inflammatory action	Khandavilli and Panchagnula. (2007)
Dendrimers	Phenylpropanoids	8-Methoxypsoralene	Gastrointestinal side effects and complications	The divergent method	Topical application	Enhanced skin permeation and concentration of 8-Methoxypsoralen in epidermis and dermis	Borowska et al. (2012)

### Improve Permeability

#### Liposomes

Liposomes are vesicular nanosystems with discrete aqueous space that enclosed by one or more concentric lipid bilayers. Liposomes can interact with epidermal keratinocytes and lipids, which leads to the enhancement of drug retention in the skin. PUVA is commonly applied for the clinical management of severe, recalcitrant psoriasis. However, the poor skin deposition and weak skin permeability of psoralen hinder its therapeutic effect in psoriasis. Therefore, Doppalapudi et al. (Doppalapudi et al., 2017) developed psoralen-loaded liposomal nanocarriers via the thin film hydration method, which bind this nanocarrier with gels to improve its skin adhesion properties and water holding capacity. The psoralen solution was completely retained in the upper SC, whereas the prepared liposomal gels penetrate across the barrier of the SC. As the liposomes interact with the skin, the liposome bilayer structure may mix with intracellular lipids in the SC, which may swell the SC intercellular lipids without transforming the multiple bilayer structure, thereby enhancing permeation (Verma, 2003). The PASI score of psoriasis mice was reduced to 1.5 after psoralen solution treatment, whereas it was reduced to 1 after psoralen liposome gel treatment. Liposome gel showed stronger efficacy, which may be due to the enhancement of the permeability, which allows the drug to reach the dermis of psoriasis skin to reduce the chemotactic activity of inflammatory cells (mononuclear and neutrophils) and the expression of inflammatory factors (such as IL-17 and IL-22) (Ternowitz, 1987).

#### Ethosomes

Ethosomes are flexible vesicles that are composed of phospholipids, water, and ethanol (usually about 20–50% of ethanol content) (Chacko et al., 2020). Ethosomes target deeper skin layers, with less initial skin deposition but greater long-term deposition when compared with traditional liposomes (Zhang et al., 2014b). This may be because the ethanol in the ethosomes can bind to the polar functional group of lecithin molecules of skin and reduce the melting point of lipids in the SC, thus increasing the lipid fluidity and cell membrane permeability. Additionally, the excellent flexibility and deformability of the ethosomes may account for their ability to squeeze across skin channels that are smaller than the vesicle diameter (Zhang et al., 2014c).

Ethosomes and liposomes were prepared for the transdermal delivery of psoralen and were evaluated for permeability and safety (Zhang et al., 2014a). The psoralen transdermal flux and skin deposition of ethosomes were respectively 3.50 and 2.15 times higher than those of liposomes. However, ethosomes on deep skin were less safe than liposomes but safer than ethanol solution. Unlike psoriasis vulgaris, purulent psoriasis is characterized by excessive infiltration of inflammatory cells into the dermis, where neutrophil protease can also activate IL-36, leading to hyperkeratosis, the thickening of the epidermis, mixed inflammatory cell infiltration, and the elevated expression of the chemokines (CCL2, CXCL6, CCL7, and CXCL2) (Johnston et al., 2016). Therefore, encapsulating drugs in ethosomes to mediate inflammatory cells in the dermis may treat pustular.

#### Niosomes

Niosomes are synthetic bilayer vesicles constituted by non-ionic surfactants and are structurally similar to liposomes (Chen et al., 2019). They seem to show great potential in transdermal delivery due to increasing the residence time of drugs in the SC and epidermis, thereby increasing local concentrations of drugs and reducing systemic absorption (Sinico and Fadda, 2009).

Celastrol, a triterpenoid extracted from Tripterygium, has significant anti-psoriasis activity. However, its poor water solubility and low permeability hinder its application in psoriasis. Meng et al. (Meng et al., 2019) developed celastrol niosome using the thin film hydration method and probe sonication for psoriasis treatment. The niosome comprised Span 20, Span 60, and cholesterol (weight ratio, 3:1:1) with an average particle size of 147 nm. Moreover, hydrogel was added as a basic carrier to prolong the residence time of the topical drug on the skin and maintain SC hydration. The *in vitro* permeation study showed the drug content of celastrol niosome hydrogels in the skin was approximately 13-fold higher than that of celastrol hydrogels. Compared with hydrogel alone, hydrogel based on celastrol niosomes had lower PASI scores and fewer white scales and erythema in psoriasis lesions. This could be partially because niosomes can enhance the fluidity of lipid and bind to keratin filaments, thereby improving the permeability and content of celastrol in the skin. The celastrol might inhibit the proliferation of keratinocytes and decrease the levels of inflammatory cytokines (IL-2, IL-1, IL-22, and IFN-γ), thereby reducing the production of chemokines and the aggregation of inflammatory cells in the epidermis.

#### Lipospheres

Lipospheres are lipid-based self-assembled systems composed of an aquaphobic, solid, lipid core coated by a layer of phospholipid molecules. Lipospheres can permeate in deeper skin, release slowly, and show good skin compatibility.

Thymoquinone showed good anti-psoriatic activity, although its clinical application is hampered by its hydrophobicity, poor water solubility, pH, and light-sensitive nature. Therefore, Jain et al. (Jain et al., 2017) prepared liposphere-loading thymoquinone with a particle size of 70 nm for the topical treatment of psoriasis. The *in vitro* drug release study showed that thymoquinone liposphere exhibited a sustained release profile for 24 h, whereas the thymoquinone solution reached release equilibrium after 6 h. The dermal distribution showed that the drug content in the epidermis and dermis was higher with thymoquinone liposphere than thymoquinone solution, whereas the PASI score was lower for thymoquinone liposphere than for thymoquinone solution. The treatment of the liposphere exerted better efficiency than the solution in reducing the expression levels of IL-17 and TNF-α. This could be explained by the fact that the liposphere, as a drug delivery system, provides a lipid environment for the drug with a small particle size, thus improving the solubility of thymoquinone and allowing it to enter the dermis through the SC barrier. Recently presented data indicate the existence of T cells that secrete IL-17 and IL-22 in the dermis. Therefore, thymoquinone lipospheres may reduce the expression of IL-17 and IL-22 in the dermis, thus reducing the proliferation and differentiation of keratinocytes and the secretion of cytokines and chemokines, which then inhibit the immune cells that produce IL-17 and IL-22, thus forming an immune cycle.

Moreover, the liposphere is usually combined with a gel to further improve adhesion and hydration. Tacrolimus is a commonly used immunosuppressive drug that treats psoriasis by inhibiting calcineurin, whereas curcumin has multiple potential targets in treating psoriasis. Hence, Jain et al. (Jain et al., 2016) combined tacrolimus and curcumin into a loaded liposphere gel to treat psoriasis through different mechanisms for an improved therapeutic effect. On the sixth day after the treatment, the erythema, scaling, and skin thickening of skin lesions can be significantly improved. The expression of TNF-α, IL-17, and IL-22 was reduced by the use of liposphere gel. This may be because the liposphere increased the permeability of the drug, allowing it to enter the SC and, thus, exert an anti-psoriasis effect.

#### Solid Lipid Nanoparticles and Nanostructured Lipid Carriers

Solid lipid nanoparticles (SLNs) are nanocarriers composed of biodegradable lipid materials. Nanostructured lipid carriers (NLCs) are the second generation of lipid nanoparticles, which are designed to overcome the drug leakage problems of SLNs, improving their physical stability. Moreover, a monolayer film may form after NLC application, thus avoiding water loss from the epidermis and increasing skin hydration. Thus, NLCs exhibit an advantage in the treatment of psoriasis.

Capsaicin, an important alkaloid from chili peppers, exerts significant inflammatory effects and has good potential for the treatment of psoriasis. However, topical application of capsaicin may cause adverse effects, including stinging, burning, and tingling, which limit their use in psoriasis treatment. Agrawal et al. (Agrawal et al., 2015) prepared capsaicin SLNs and NLCs via a solvent diffusion method. The prepared SLNs and NLCs exhibited average particle sizes of 182.5 and 145.3 nm, respectively. Compared with the blank capsaicin solution and SLNs, NLCs had better skin permeation and skin retention. This result may be explained by the fact that NLCs have a higher loading capacity, lower water content, smaller particle size, and greater lipophilicity, which enhance the occlusive effect and the tightness of junctions between the drug and SC. Fang et al. (Fang et al., 2008) also prepared NLCs and SLNs for the topical delivery of psoriasis. A similar conclusion was drawn: compared with SLNs, NLCs have better drug permeability. Therefore, compared with SLNs, NLCs are the better selection for transdermal delivery of natural products for the treatment of psoriasis.

#### Nanoemulsions

Nanoemulsions are thermodynamically unstable systems stabilized by an interfacial layer of surfactant and cosurfactant. The elastic properties and fluid performance of nanoemulsions may contribute to their better permeability through SC.

Curcumin, a natural product isolated from plants, has good potential in the treatment of psoriasis. However, its poor solubility and skin permeability are a major obstacle in the treatment of psoriasis. For the topical treatment of psoriasis, Algahtani et al. (Algahtani et al., 2020) formulated curcumin nanoemulsion with a particle size of 10.57 nm using a low-energy emulsification method. Moreover, the prepared nanoemulsion was combined with polymeric hydrogel using carbopol 934 as a gelling agent to hydrate the SC and improve the ductility of curcumin. Using nanoemulsion gel, the amount of the drug deposited in the skin and the cumulative amount of the drug that permeated were respectively almost 7 and 5 times those of curcumin gel. As the drug is negatively charged and has a small particle size, the permeability of the drug can be enhanced (Baspinar and Borchert, 2012). Therefore, negatively charged curcumin nanoemulsion with a particle size of 10.57 nm can also improve the permeability of drugs in the skin (Khurana et al., 2013). Compared with curcumin gel and betamethasone-17-valerate gel, treatment with curcumin gel in IMQ-induced psoriasis mice led to more improvement in psoriatic signs after 4 days. This may be because curcumin-loaded nanoemulsions can penetrate across the epidermal basement membrane and inhibit the expression of inflammatory factors, thereby inhibiting keratinocyte proliferation.

Paclitaxel is effective in the treatment of psoriasis. However, paclitaxel is a macromolecular drug with strong lipid solubility, poor water solubility, and poor permeability, which hamper its clinical adoption. Nanoemulsions are proper carriers that can deliver paclitaxel into and across the skin because of their interaction with SC. Additionally, because of its lipid solubility and macromolecular characteristics, paclitaxel nanoemulsion may be capable of localizing in deeper skin layers without entering systemic circulation through the dermis, thus reducing the systemic escape. The oral paclitaxel bioavailability was over 70% after the administration of paclitaxel-loaded nanoemulsion (Pandey et al., 2020). Erythrodermic psoriasis is a severe type of psoriasis with systemic symptoms (fever, chills, and headache), with skin lesions covering the whole body. To improve efficacy by inhibiting epidermal proliferation and because of its anti-inflammatory capabilities, paclitaxel nanoemulsion combined with oral administration can be considered for the treatment of erythrodermic psoriasis.

#### Nanospheres

Nanospheres may be defined as a matrix system in which a drug is dissolved, encapsulated, and chemically bound or adsorbed in a polymer matrix (Letchford and Burt, 2007). Nanospheres have unique potential in the treatment of skin diseases, especially the appearance of tyrosine-derived nanospheres, because of their low critical aggregation concentration and ability to target hair follicles (Sheihet et al., 2008; Batheja et al., 2011).

Kilfoyle et al. (Kilfoyle et al., 2012) developed tyrosine-derived nanospheres of paclitaxel to allow skin drug localization. The paclitaxel solubility of TyroSpheres was about 4000-fold more than that of phosphate-buffered saline solution. Moreover, the paclitaxel of TyroSpheres demonstrated a sustained, dose-controlled release for more than 72 h when mimicking skin permeation conditions. The TyroSpheres were mainly distributed in the epidermis and were found less in the dermis. This discrepancy could be attributed to the richness of lipids in the SC, as the macromolecular compound of paclitaxel may stay in the SC and slowly penetrate the lower, viable epidermis. Therefore, paclitaxel TyroSpheres may mainly concentrate in the basal layer of psoriatic skin and may inhibit the keratinocyte hyperproliferation in psoriatic skin lesions.

#### Foams

**FIGURE 4:**
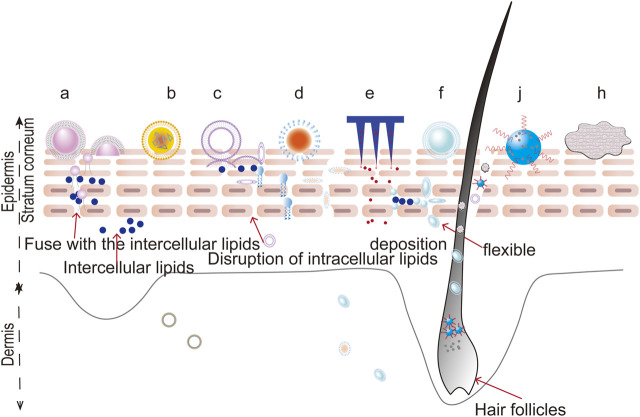
The potential penetration routes of novel drug delivery systems. a: liposomes, b: nanostructured lipid carriers, c: niosomes, d: nanoemulsions, e: microneedles, f: ethosomes, j: nanocrystals, h: foams.

Foams are defined as colloidal solutions, where gas is dispersed in a liquid, solid, or gelled matrix. Compared with traditional formulations (such as ointments, creams, lotions, gels, and solutions), foams show distinct advantages as novel topical carriers due to their low irritancy potential, uniform spreading, lack of residual oil, and non-stickiness. Recently, foams have shown potential in the treatment of psoriasis. The properties of foams allow a drug to be quickly broken down on the scalp and get into the SC through the roots of the hair, which is suitable for treating scalp psoriasis (Franz et al., 1999; Feldman et al., 2000; Feldman et al., 2001). The rapid evaporation of the foam propellant results in an increase in the surfactant concentration in the remaining foam, thereby increasing the permeability. It can also be considered for topical treatment of psoriasis in natural products, including oxymatrine and capsaicin, both to increase permeability and to reduce irritation (Puig and Carretero, 2019; Velasco et al., 2019; Lebwohl et al., 2020). The potential penetration routes of novel drug delivery system is shown in Figure 4.

### Improve the Targeting Ability

#### Modified Ethosomes

When ethosomes are conjugated with ligands, their targeting ability can be effectively improved. For example, HA is the natural ligand of CD44 protein (a highly expressed protein in psoriatic skin). Hence, HA can be combined with ethosomes to target CD44 protein for psoriasis (Lindqvist et al., 2012). Zhang et al. (Zhang et al., 2019) prepared curcumin ethosomes modified with HA for the targeted treatment of psoriasis. Compared with plain ethosomes and propylene glycol solution, applying HA ethosomes as delivery vehicles led to significant enhancements in the cumulative transdermal amount of curcumin and the amount retained in the skin. Two lines of evidence offer possible explanations. On the one hand, HA shows a dense, three-dimensional network structure, improving water absorption and moisture preservation and thus increasing the hydration of the SC and improving the drug permeability. On the other hand, the specific binding of HA to CD44 in the skin may lead to the enhancement of drug retention in the skin. The curcumin HA-ES group had the lowest PASI score, and the expression of CCR6, IL-17A, and IL-17F was also decreased.

Curcumin possesses a tremendous therapeutic potential to treat psoriasis, whereas its poor percutaneous penetration restricts its clinical application. Thus, composite phospholipid ethosomes were prepared by Li et al. (Li et al., 2020) to improve the permeability of curcumin. The mixture of unsaturated phosphatidylcholine and saturated hydrogenated phosphatidylcholine (1:1) was used as a composite phospholipid carrier to improve the stability of an unsaturated phospholipin. The permeability of curcumin in composite phospholipid ethosomes was significantly higher than that of free curcumin. The fluorescence microscopy imaging results showed that phospholipid ethosomes could effectively deliver curcumin to the deeper layers of the skin. Moreover, after the application of phospholipid ethosomes, the curcumin could be observed in the hair follicles, especially hair roots. This suggests that the hair follicle pathway is one of the percutaneous pathways of curcumin-composite phospholipid ethosomes. Ethosome-encapsulated curcumin provides the opportunity to treat scalp psoriasis by targeting hair follicles, thereby reducing the aggregation of T cells, macrophages, and neutrophil inflammatory cells in hair follicles.

#### Microneedles

Microneedles are Biomedical Micro-Devices composed of arrays of fine needles, which can painlessly puncture the skin by avoiding the stimulation of nerve endings. Microneedles can effectively deliver a drug across the SC and show promising activity for further application in psoriasis (Gujjar et al., 2015; Tekko et al., 2020). Paleco et al. (Paleco et al., 2014) prepared lipid microparticles of quercetin to improve its stability and solubility and then combined them with microneedles to further improve permeability. The permeability of free quercetin in the skin applied with microneedles was not significantly improved. Although the application of microneedles provides an effective route for percutaneous penetration by disrupting the skin barrier, the permeability is hampered by quercetin’s poor solubility. However, the permeability is greatly improved by the combination of microneedles and lipid microparticles. Compared with untreated skin, the lipid microparticles localized in the SC and viable epidermis increased by 2 and 5 times, respectively, after treatment with microneedles. This can be explained by the fact that the microparticles can penetrate into the epidermal area through microconduits produced by the microneedles, but do not spread beyond the epidermis to the deeper layers of skin. These results indicate that the combination of quercetin lipid particles and microneedles has a certain targeting effect on the epidermis. Therefore, quercetin combined with microneedles can be applied to treat psoriasis by reducing the expression of IL-17 and TNF-α (Chen et al., 2017a), thus reducing the infiltration of inflammatory cells in the epidermis. For poorly soluble drugs, microneedles should be combined with nanocarriers, such as liposomes, ethosomes, nanoparticles, or nanoemulsions, to achieve better therapeutic effects. Recently, microneedle-based electrochemical devices have been widely applied to sense biomarkers in the skin tissue fluid (Madden et al., 2020a). Therefore, the combination of microneedles and particular electrochemical devices can be designed to sense inflammatory factors or cells, consequently achieving an accurate diagnosis of the disease location.

#### Nanocrystals

Nanocrystals typically consist of pure drugs and stabilizers, creating a carrier-free nanoparticle system. Nanocrystals have the advantage of high drug loading, as well as a prolonged adhesion time. Percutaneous therapy of nanocrystals could be expected to achieve an anti-psoriasis effect by targeting hair follicles. Pelikh et al. (Pelikh et al., 2020) prepared curcumin nanocrystals by a bead milling method to target hair follicles. The enhanced permeability could be due to the prepared curcumin nanocrystals penetrating the hair follicle at a mean depth of 271 μm, close to the lower part of the infundibulum of the hair follicles, where the SC is relatively weak. In this area, the drug absorption is efficient, which means the dissolved drug substances can passively spread to deeper layers of the skin and thus continue to flow into the viable dermis. On the one hand, the high drug loading of nanocrystals can lead to more drugs reaching the hair follicles. On the other hand, nanocrystals can serve as a depot, where the drug can remain in the hair follicles for days or weeks and result in long-lasting drug delivery. Therefore, by targeting hair follicles, curcumin nanocrystals can be considered for the treatment of scalp psoriasis. Other nanoformulation-based natural products for the treatment of psoriasis are shown in Table 6.

**TABLE 6:** The advantages/disadvantages of nanoformulation for the treatment of psoriasis.

Nanoformulation	Advantages	Disadvantage
Liposomes	Biocompatible, ease of surface modification and can improve the drug retention in the skin	Rapid drug leakage, large size
Ethosomes	Can target deep skin layers, show excellent flexibility and deformability	Risk of organic solvent residue
Niosomes	Can increase the residence time of drugs in the SC and epidermis	Physical instability
Lipospheres	Can release slowly, and show good skin compatibility	Physical and chemical instability
SLNs	Biodegradable and biocompatible	Physical instability, rapid drug leakage
NLCs	Higher drug loading capacity and stability compared to SLNs	—
Nanoemulsions	Elastic properties and fluid performance	Usage of expensive instruments
Nanocrystals	High drug loading capacity	Difficult to be surface-modified

## Prospects of Natural Product-Based Novel Drug Delivery Systems for the Treatment of Psoriasis

Natural products have obvious advantages in the treatment of psoriasis, especially since the combination of novel drug delivery systems and natural products provide a potentially effective therapeutic strategy for psoriasis. However, some problems are remaining. The major factor leading to debate is safety issues. In psoriatic lesions, the drug absorption pathway, skin accumulation, and systemic circulation have been significantly changed compared with normal skin. The delivery capacity, efficacy, and safety of a novel drug delivery system should be considered comprehensively to achieve a better therapeutic effect. Until now, most of the natural product for psoriasis is single natural product, which can not achieve optimal therapeutic. Hence, when the condition is more complex, such as moderate or severe psoriasis, natural product can exert synergistic effect with other biologic agents but the joint mechanisms of action need to be carried out systematically. Additionally, the limitations of novel drug delivery systems, including low drug loading, physicochemical properties and stability, encapsulation efficiency and industrial production challenge, have hindered the application in clinical trials. Therefore, further studies should focus more attention on these factors to guide their effective therapy for psoriasis. As for the current treatment of psoriasis with natural products combined with novel drug delivery systems, available data on their clinical effectiveness are limited because of most studies being at the preclinical research stage with a single-animal model. Moreover, the existing treatments are mainly for mild or moderate psoriasis rather than for severe psoriasis. The era of precision medicine sets a higher requirement for the treatment of psoriasis. Perhaps the future management of psoriasis can advance in the direction of targeted therapy and precision medicine by targeting specific cells or genes.

## Abbreviations

cfDNA, cell-free DNA; DC, dendritic cells; CCL2, chemokine ligand 2; DMSO, dimethyl sulfoxide; IFN, interferon; IL, interleukin; JAK/STAT, Janus kinase–signal transducers and activators of transcription; LPS, lipopolysaccharide; MAPK, mitogen-activated protein kinase; MDC, myeloid dendritic cell; mRNA, messenger RNA; NET, neutrophil extracellular trap; NF-κB, nuclear transcription factor κB; NLC, nanostructured lipid carriers; pDC, plasma cell-like dendritic cell; PUVA, psoralen ultraviolet A; SLN, solid lipid nanoparticles; SP, substance-P; STAT, signal transducers and activators of transcription; SC, stratum corneum; TNF, tumor necrosis factor; TLR, toll-like receptor; UVA, ultraviolet A; VEGF, vascular endothelial growth factor.
